# Quantitative Assessment of Woven Fabric Surface Changes During Martindale Abrasion Using Contactless Optical Profilometry

**DOI:** 10.3390/ma18153636

**Published:** 2025-08-01

**Authors:** Małgorzata Matusiak, Gabriela Kosiuk

**Affiliations:** 1 Lodz University of Technology, Faculty of Material Technologies and Textile Design, Institute of Architecture of Textiles, 90-924 Lodz, Poland; 2 Institute of Security Technologies MORATEX, 90-505 Lodz, Poland

**Keywords:** abrasion, woven fabrics, profilometer, angle distribution, autocorrelation function

## Abstract

The abrasion resistance of fabrics is one of the basic properties determining the utility performance and durability. The abrasion resistance of textile materials is measured using the Martindale device according to appropriate standards. The sample breakage method is the most commonly used of the three methods. The method is based on organoleptic assessment of fabric breakage. The method is time-consuming, and results may be subject to error resulting from the subjective nature of the assessment. The aim of the presented work was to check the possibility of the application of contactless 3D surface geometry measurement using an optical profilometer in an assessment of changes in fabrics’ surface due to the abrasion process. The obtained results confirmed that some parameters of the geometric structure of fabric surfaces, such as the highest height of the roughness profile Rz, the height of the highest pick of the roughness profile Rp, the depth of the lowest valley of the roughness profile Rv, the depth of the total height of the roughness profile Rt, and the kurtosis Rku, can be used to assess the abrasion resistance of fabrics. It is also stated that using the non-contact optical measurement of fabric surface geometry allows for an assessment of the directionality of surface texture. For this purpose, the autocorrelation function and angle distribution function can be applied.

## 1. Introduction

Textile products are not a monolith, but a collection of fibers that are connected in various methods [1]. As a result, abrasion resistance is one of the fundamental properties that determine a textile material’s utility, performance, durability, and overall lifespan. Abrasion resistance often determines withdrawal from use for clothing, upholstery, carpets, and rugs, as well as bed linens.

When the surface of a textile product undergoes rubbing, one of the following phenomena may occur [2]:

- Pulling out of the unclamped end of the fiber;
- Partial pulling out of the fiber, with simultaneous loosening of the cohesion of the fibers in the product;
- Deformation of the fiber within the limits of elasticity;
- Plastic deformation of the fibers, equivalent to loosening of the cohesion of the fibers in the product;
- Tearing of the fibers.

In the abrasion process of textile materials, the yarn properties—particularly the twist and its type—play a crucial role [2,3,4]. A high yarn twist results in fibers adhering tightly to one another, forming a cohesive, almost monolithic structure. Fabrics made from such tightly twisted yarns generally exhibit greater resistance to abrasion compared to those made from yarns with a lower twist. However, these fabrics often possess less desirable qualities: they tend to be stiffer and offer reduced thermal insulation due to the limited air trapped between the fibers.

Yarn thickness uniformity also significantly affects a fabric’s abrasion resistance. When yarns are uniform in thickness, pressure is evenly distributed across all yarns in contact with the abrasive surface. Conversely, if the fabric is made from yarns of varying thickness, the abrasive surface primarily contacts the thicker segments. These thickened areas endure concentrated abrasive forces, leading to greater fiber loss and an increased risk of yarn breakage at those points.

The abrasion resistance of fabrics also depends on the density of the warp and weft, as well as the way by which the yarns are interlaced into the fabric, i.e., the weave [3]. With the same number of threads per unit length, weaves with a higher number of interlacing points per unit length provide better fiber attachment to the yarn than weaves with fewer interlacing points per unit length.

Longer interlacing points, on the other hand, create a larger contact area with the abrasive surface and, consequently, influence abrasion resistance. Long floats in a weave are more exposed and will abrade faster, leading to yarn breakage. For instance, satin weave fabric will abrade more easily than a twill weave [5].

Another important factor influencing the abrasion resistance of woven fabrics is the degree to which the outer surfaces of the yarns in both the warp and weft systems are elevated above the fabric’s mid-plane. When the outermost points of the yarn lie on one plane, the abrasive pressure is evenly transferred by the two yarn systems. Fabrics that meet this condition typically exhibit greater durability compared to those with a similar weave structure but lacking uniform yarn alignment on a common surface.

Radmanovac et al. investigated the abrasion resistance of woven fabrics used in protective clothing [6]. They confirmed that the abrasion resistance of woven fabrics is influenced by several factors, such as raw material composition, yarn type (single or twisted), weave, type of finishing treatment, fineness, and type of abrasive material. On the basis of the obtained results, they stated that fabrics made from twisted yarns, blends of cotton and polyester fibers, finer yarns, and yarns in plain weave are characterized by higher resistance and exhibit less deformation or damage due to wear, compared to other fabrics tested.

Das [7] also demonstrated the influence of yarn and fabric construction on the abrasion resistance of woven fabrics. He investigated soft silk fabrics produced from bivoltine cocoons in both dry and wet states subjected to 500 and 1000 abrasion cycles. The results revealed a reduction in the bursting strength of soft silk fabrics in dry and wet states. The study also noted a higher reduction in the wet state. The study also demonstrated that the abrasion resistance of silk fabrics can be improved by increasing the geometric area of contact between the fabric and the abradant. This can be achieved by using a higher cover factor and ensuring equal crown heights in both warp and weft yarns.

Anyab et al. [8] evaluated denim woven fabrics in terms of their abrasion resistance. They observed that the abrasion resistance changes with changes to several parameters of denim fabric, including fiber and yarn type, sizing, desiring, shedding mechanism, and finishing processes.

Scientific works published to date on the abrasion resistance of woven fabrics primarily focus on the relationship between the abrasion resistance and structural parameters of woven fabrics. Most of the existing research investigates specific categories of fabrics, such as denim, silk-made fabrics, and fabrics for protective clothing. Within the investigated group of fabrics, researchers have identified various factors that influence abrasion resistance. It is important to note that abrasion resistance is closely linked to the quality of the fabric surface, as abrasion primarily affects the outer layer, i.e., the surface. All analyzed factors, such as raw material composition, yarn type and parameters, weave, and warp and weft count, influence most fabric properties, including abrasion resistance. However, there is a noticeable lack of research focused on fabric surface quality, especially surface topography, and its effect on fabric behavior during abrasion. The geometric structure of woven fabrics is determined by parameters such as weave, yarn thickness and uniformity, hairiness, and warp and weft density. Equally important are the tension of warp and weft threads during weaving and their relaxation in the finishing process. It determines the so-called phase of woven fabrics [9,10], which refers to the degree and regularity of the elevation of yarns of both systems above the middle plane of the fabric. This aspect is crucial in understanding abrasion behavior, as the elevated points on the fabric surface are the first to come into contact with the abrasive material and are therefore the first to wear away. A precise assessment of the fabric surface structure could significantly aid in understanding the behavior of the fabrics during the abrasion process and in modelling their abrasion resistance.

The abrasion resistance of textile materials is commonly measured using the Martindale device in accordance with the standards [11,12]. Abrasion resistance refers to the resistance of a fabric to repeated friction with other materials. There are three methods of assessment of fabric abrasion resistance using the Martindale test: the specimen breakage method, mass loss method, and appearance change method [11,12,13,14]. Among these, the sample breakage method is the most widely used due to its small error and straightforward, intuitive results, which make it easy to compare the wear resistance of different fabrics. The assessment of the mass loss method and appearance change method is more complex, but it can reflect the wear resistance of samples at different friction stages [14]. The Martindale method is applicable for testing the abrasion resistance of fabrics, carpets, rugs, leather, and other soft materials.

As previously mentioned, the breakage method is subjective, and as a result, its outcomes may be prone to errors arising from the subjective nature of the assessment. It would be beneficial to develop an objective method for assessing changes to the fabric surface after abrasion tests—ideally by numerically describing alterations in surface geometry. This can be achieved by 3D non-contact measurements that use optical methods to analyze the geometric structure of a textile surface. In general, surface geometry measurement methods fall into two categories: contact and non-contact. In contact methods, the stylus physically touches the surface of the object being measured and moves along the surface. The vertical displacement of the stylus is converted into an electrical signal, which can be recorded as a profilograph and processed further to yield numerical values of roughness parameters. However, contact methods allow for measurement in two dimensions, recording the height at each point along the line. In contrast, non-contact methods use optical instruments to measure the surface in three dimensions, generating data across the entire area. These methods are significantly more accurate than contact methods, largely because in contact methods, the dimensions of the measuring tip (stylus) determine the irregularities detected by the instrument. Irregularities smaller than the tip dimensions cannot be detected, which means that the recorded profile may differ significantly from the actual surface topography. Optical methods, by contrast, can capture even the smallest recesses on the surface, providing a much more accurate and faithful representation of its geometry.

In the measurement of solid objects, such as in mechanical engineering, these methods are commonly used to assess the surface wear of machine components, such as bearings. Wear is a progressive modification of a surface. It is not limited to material removal. Depending on the wear mechanism, material may also be redistributed, or even added. Surface roughness measurement using an optical profilometer can be employed to quantify the wear effects. Data from a 3D optical surface measurement allow for detailed analysis of wear scars, including their depth, width, volume, and texture, as well as changes in surface roughness and other wear-related characteristics.

Similar surface changes occur on the fabric surface during the Martindale abrasion test. These changes can be evaluated using 3D non-contact surface topography measurement methods. Optical profilometry enables acquisition of a wide field of view of the fabric’s surface, allowing for precise measurement and analysis of parameters that characterize the geometric structure of the fabric’s surface. Research in this area is already underway. Ceven and Özdemir [15] evaluated abrasion resistance based on the relative difference between images of areas covered by chenille pile before and after abrasion cycles. In a patent [16], a novel method for assessing abrasion resistance is based on image brightness level before and after abrasion cycles. This method is specifically dedicated to metal-coated textile fabrics. Leucker [17] explored a method for the abrasion resistance of light-mass polypropylene nonwovens using images of samples being binarized using threshold values established in a prior study on mass loss due to abrasion. Jasińska [4] proposed a comprehensive analysis of visible changes in the sample’s image profile to establish endpoint detection criteria that align with standard requirements. Her method, based on image analysis techniques, allows for identifying the endpoint of the abrasion test without the need for constant manual inspection of a sample surface. The method presented for abrasion resistance assessment involves evaluation of brightness profiles obtained from scans of samples.

The aim of present study was to evaluate the feasibility of using 3D non-contact surface geometry measurements, obtained via an optical profilometer, to assess the changes in fabric surfaces resulting from the abrasion process. Measurement of surface geometry and its changes was conducted using the MicroSpy^®^ Profile profilometer, manufactured by FRT GmbH, Bergische Gladbach, Germany [18,19,20]. The method enabled the analysis of various parameters and functions characterizing the geometric structure of the investigated fabric surface.

The novelty of this paper lies in the introduction of objective surface geometry measurement using a non-contact 3D optical profilometer for the assessment of abrasion resistance of woven fabrics. It is proposed that 3D surface measurement using optical methods has the potential to support—or replace—the conventional subjective breakage evaluation method. Additionally, these measurements may enable the modeling of fabric abrasion behavior based on surface geometry.

## 2. Materials and Methods

**Figure 1:**
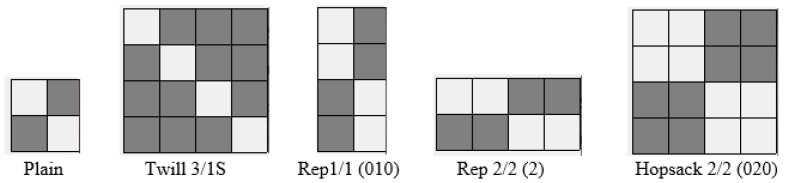
Repeats of weaves applied in the investigated woven fabrics.

Cotton woven fabrics were the objects of analysis. They included fabrics of different weaves (Figure 1): plain, 3/1S twill, 1/1 (010) rep, 2/2 (2) rep, and 2/2 (020) hopsack. Other structural parameters, such as the linear density of the warp (50 tex) and weft (100 tex), as well as the number of picks and ends, were kept practically constant. Minor variations in structural parameters resulted from relaxation during the finishing process. The fabrics were specifically designed and manufactured in Lodz University of Technology for the research purposes. The basic structural characteristics of the investigated woven fabrics are presented in Table 1.

The measurements of the basic structural parameters of the tested fabrics were performed in accordance with the relevant standards:

- Measurement of mass per unit area—PN-ISO 3801:1993 [21];
- Measurement of warp and weft density—PN-EN 1049-2:2000 [22];
- Measurement of fabric thickness—PN-EN ISO 5084:1999 [23].

**Table 1:** The basic structural parameters of the investigated fabrics [24].

No.	Weave	Mass per Unit Area g/m^2^	Warp Density /cm^−1^	Warp Density /cm^–1^	Thickness mm
1	Plain	292	31.2	11.5	0.67
2	Twill 3/1S	292	31.7	11.6	0.78
3	Rep 1/1 (010)	284	31.7	11.9	0.83
4	Rep 2/2 (2)	293	32.0	11.8	0.65
5	Hopsack 2/2 (020)	287	31.6	11.7	0.79

**Figure 2:**
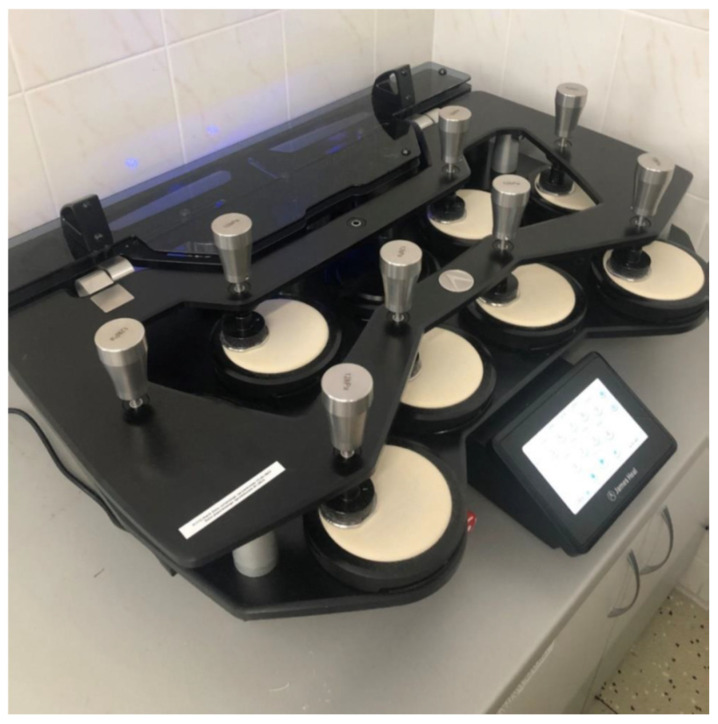
The Martindale tester applied in the presented work.

The abrasion process was conducted using the Martindale device (model 1609 MAXI_MARTINDALE) manufactured by James Heal (Halifax, UK) (Figure 2). During the test, a circular fabric sample, secured in a holder, moves in a translational motion following a Lissajous curve path. Simultaneously, the sample holder rotates freely around its axis, and the fabric is abraded by the abrasive material. This method involves subjecting the fabric to cyclic friction generated by an electrically driven abrasive medium. The sample is rubbed against either a reference fabric or a coarse standard fabric under a defined pressure. The test is conducted either for a specified number of cycles or until a predetermined level of fabric damage is reached. The test result is expressed as the number of friction cycles that the sample has withstood or as the degree of surface wear.

**Figure 3:**
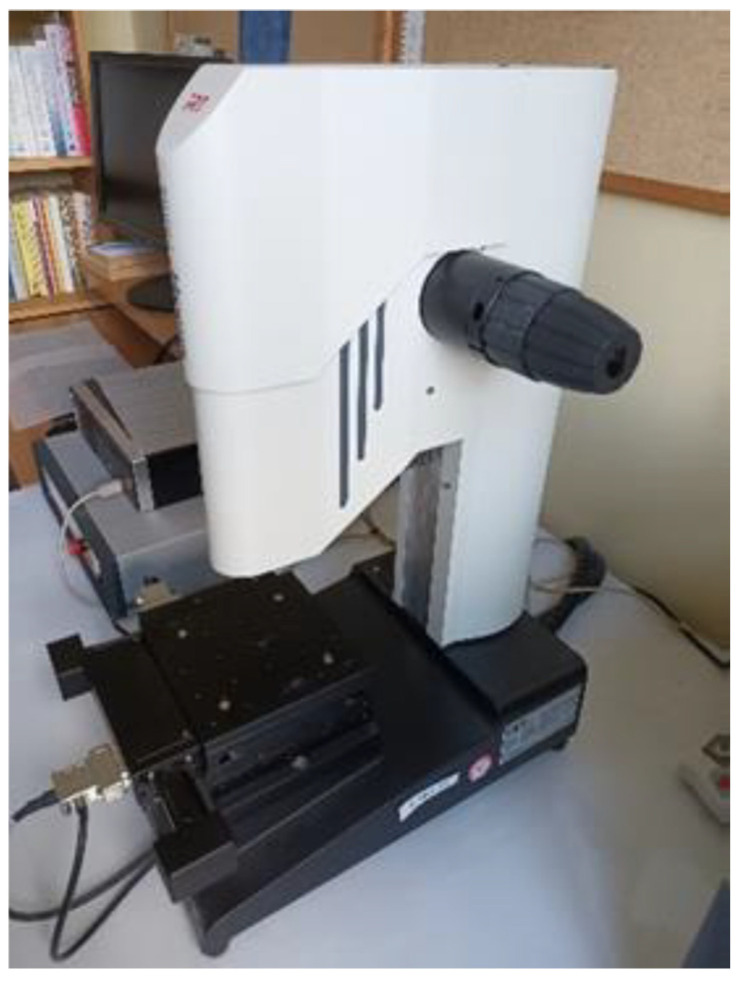
The MicroSpy^®^ Profile profilometer.

In the conducted tests, the samples were subjected to 10,000 abrasion cycles. A wool fabric compliant with the relevant standards was used as an abradant. The basic parameters of the abradant are as follows: mass per square meter—208 g/m^2^, warp density—180/cm, weft density—139/cm. The abradant was compressed while mounting on the holder with a pressing weight of 2.5 kg and a diameter of 120 mm, according to the standard [11]. Before and after the abrasion process, the surface geometric structure of the samples was measured using an optical profilometer—MicroSpy^®^ Profile by FRT (Figure 3). The instrument utilizes an optical method for precise measurement of the surface characteristics. It applies the principle of chromatic distance measurement by means of the patented CWL (chromatic white light) sensor, which makes use of the chromatic aberration of optical lenses [18].

Surface geometry measurement was performed on a square area with a side length of 15 mm. For the fabric samples before the abrasion test, the measurements were taken from five specimens, while for the samples after the abrasion test, two specimens were tested. Next, the results from the profilometer were processed using the specialized Mark III software, also developed by FRT GmbH, Berische Gladbach, Germany [25]. The parameters and functions describing the surface geometry of the investigated fabrics were calculated in accordance with the ISO 4287: 1997 standard—Geometrical product specification (GPS)—Surface texture—Profile method—Terms, definitions and surface texture parameters [26].

To eliminate the waviness component, the profilometer data were first filtered using a 1.4 mm cutoff filter [20].

The results from the profilometer were analyzed statistically using one-way and two-way ANOVA (Analysis of Variance). The parameters from the profilometer were taken as the dependent variables, while the weave and “before/after Martindale test” were taken as the main factors. The statistical significance was assessed at the 0.05 significance level.

## 3. Results

**Figure 4:**
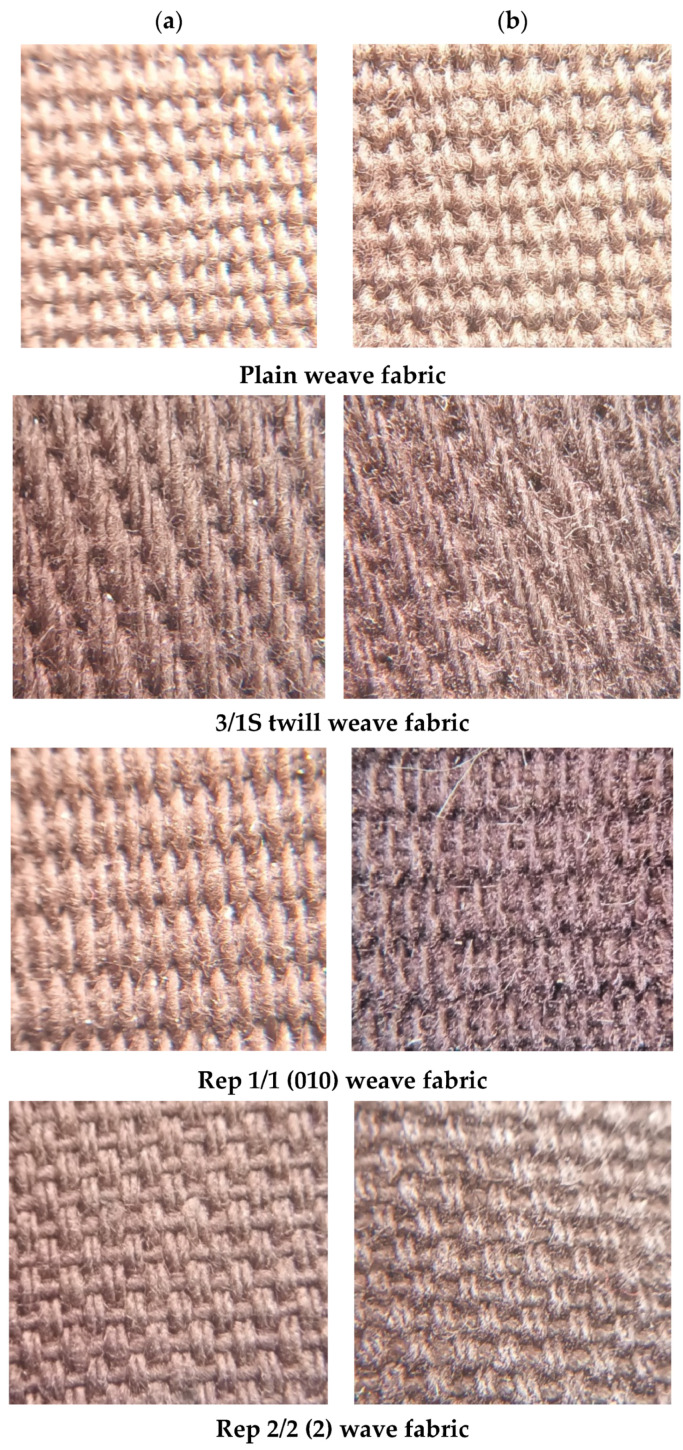

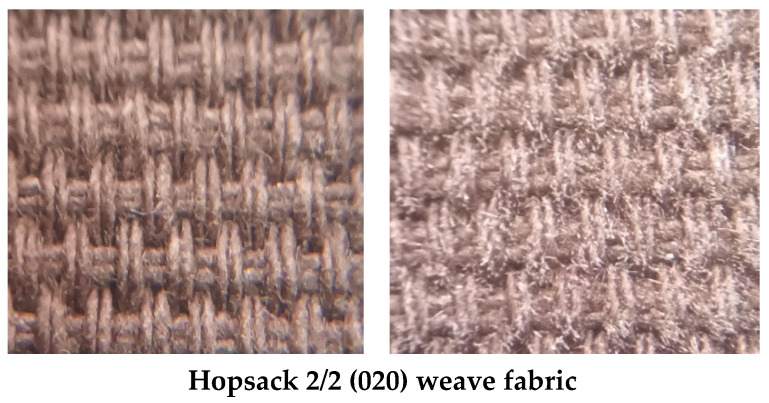
Pictures of the variants of the investigated fabrics before and after the abrasion test: (**a**) before abrasion, (**b**) after abrasion.

Exemplary pictures of the investigated woven fabrics both before and after the abrasion tests are presented in Figure 4. It can clearly be seen that the surface of the fabrics changes significantly. The most significant visual changes can be observed in the 1/1 (010) rep and 2/2(2) hopsack weave variants. The surface change that can be observed visually is primarily the appearance of loose fiber ends on the surface. This causes the surface to have a hairy appearance.

The MicroSpy^®^ Profile profilometer provides the data in the form of color pictures of the measured surface. A color scale bar on the right side of the pictures expresses the height scale. Each color represents a height of individual points on the measured area.

**Figure 5:**
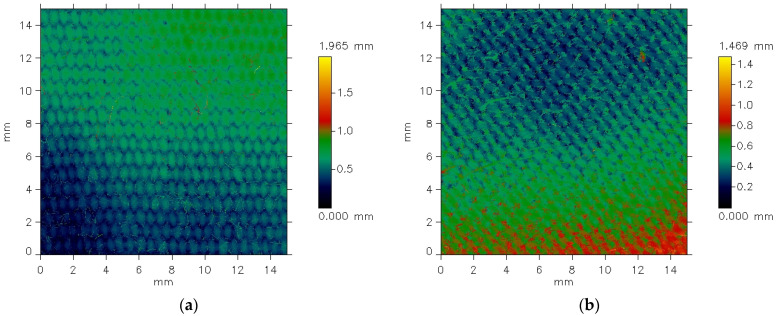
Unfiltered pictures of the plain weave fabric surface: (**a**) before the abrasion test, (**b**) after the abrasion test.

Figure 5 presents exemplary data provided by the profilometer for the plain weave fabric. The picture shows unfiltered data for the fabric both before (Figure 5a) and after (Figure 5b) the abrasion test.

**Figure 6:**
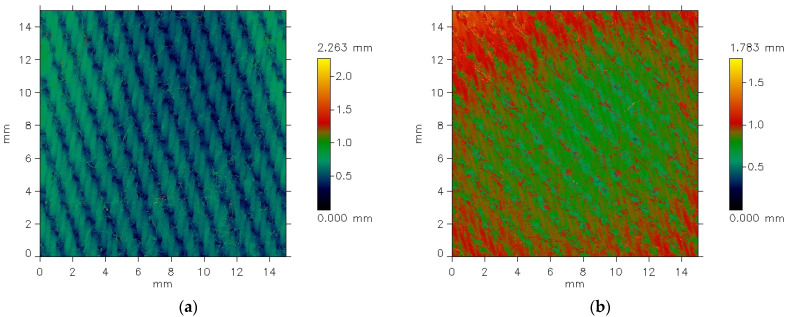
Unfiltered pictures of the 3/1S twill weave fabric surface: (**a**) before the abrasion test, (**b**) after the abrasion test.

The differences between the images are clearly visible. Before the abrasion test, the surface irregularities on the fabric surface appear regular and correspond to the weave structure. Fragments of warp threads passing over the weft threads are distinctly visible. In contrast, after abrasion (Figure 5b), the plain weave pattern is less clearly reflected. Additionally, the surface of the abraded fabric shows visible fiber fragments and ends that have been pulled from the yarn structure due to the abrasion process, resulting in a piled fabric surface. Some texture directionality can also be observed. Similar effects were noted in the other investigated fabrics. Figure 6 presents the images of the surface of the 3/1S twill weave fabric both before and after the abrasion test.

**Figure 7:**
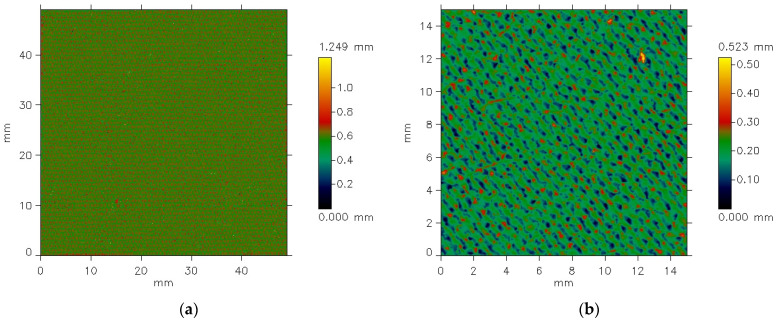
Filtered pictures of the plain weave fabric surface: (**a**) before the abrasion test, (**b**) after the abrasion test.

**Figure 8:**
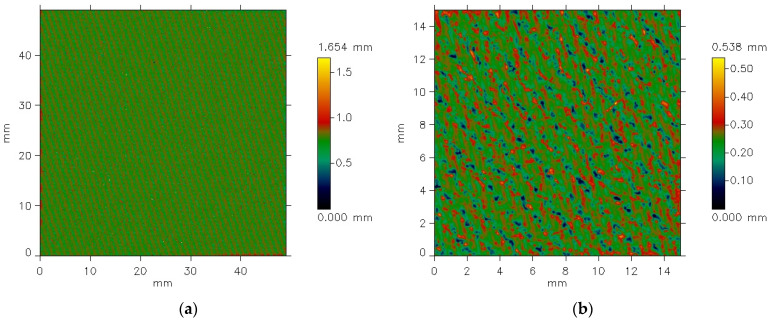
Filtered pictures of the 3/1S twill weave fabric surface: (**a**) before the abrasion test, (**b**) after the abrasion test.

The texture of the fabrics consists of two components: roughness and waviness. However, the waviness observed in the measured data is not an inherent feature of the fabric surface texture. This is an effect of imprecise adhesion of the sample to the measuring device’s table. For further analysis, it was necessary to eliminate the waviness effect. This was carried out using the cutoff filter (λc = 1.4 mm). Figure 6 and Figure 7 show the surface texture of the fabrics after waviness elimination. Similar to the unfiltered data, the weave pattern remains visible in the filtered images of the fabrics before the abrasion test. The abrasion test alters the surface texture of the fabrics, as shown in Figure 7b and Figure 8b.

Based on the profilometer data, the parameters characterizing the geometric structure of fabric surface were calculated in accordance with the ISO standard [26]. The following parameters were computed and analyzed:

Ra—an arithmetic mean of the absolute of the ordinate values within a defined area. It expresses, as an absolute value, the difference in height of each point on the measured surface and the arithmetical mean height of the surface.

Rq—a root mean square value of the ordinate values within a defined area. It is equivalent to the standard deviation of the height of all points on the measured surface.

Rz—the mean roughness depth of a surface within the sampling length. It is a sum of the Rp and Rv, where:

Rp—the maximum peak height of a surface within the sampling area,

Rv—the maximum valley depth of a surface within the sampling area.

Rt—total height of the profile, which is a sum of the maximum peak height and the maximum valley depth within the evaluation length, not the sampling length.

Rpk—reduced peak height, which is the height on the *z*-axis derived from the material-ration curve [27].

Rku—kurtosis, a parameter that relates to the tip geometry of peaks and valleys and is suitable for analyzing the degree of contact between two objects. The interpretation is as follows:

Rku = 3: The height distribution is normal,

Rku > 3: The height distribution is sharp,

Rku < 3: The height distribution is even.

The mean values of the parameters and the standard deviation (SD) are presented in Table 2 and Table 3. For the fabrics after the abrasion test, the standard deviation values are not provided due to the limited number of repetitions—only two specimens were tested for each fabric variant. The individual result for the fabric specimens after the abrasion test are available in the Supplementary Materials—Table S4.

## 4. Discussion

**Figure 9:**
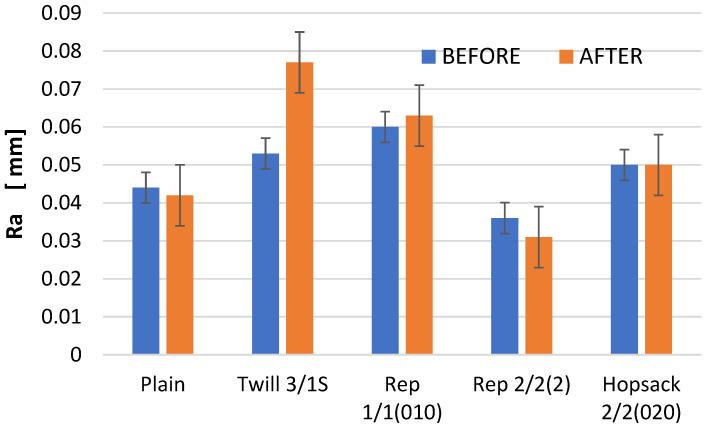
The values of the Ra parameter for the fabrics before and after the abrasion test.

Figure 9 presents a comparison of the Ra parameter values before and after the abrasion test.

**Figure 10:**
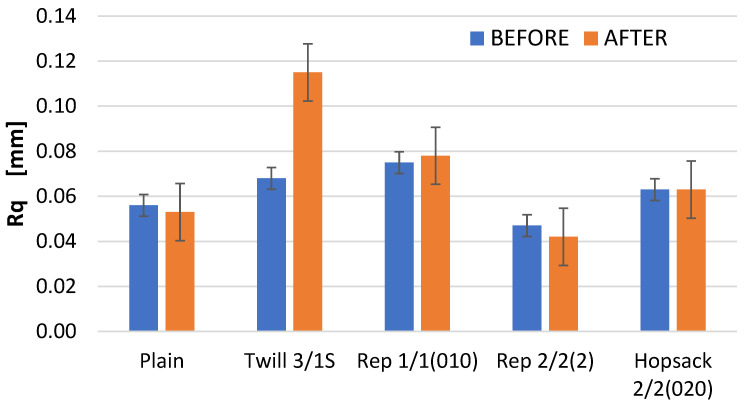
The values of the Rq parameter for the fabrics before and after the abrasion test.

Changes in the Ra parameter values due to abrasion were observed across all analyzed fabric variants; these changes were generally minor, with the exception of the fabric with the 3/1S twill weave. In two cases—the 3/1S twill and 1/1 (010) rep weaves—an increase in the Ra parameter was noted after abrasion compared to the value of this parameter before abrasion. Conversely, for the plain and 2/2 (2) rep weave fabrics, the opposite situation was noted. In the case of the 2/2 (020) hopsack weave fabric, the Ra value remained unchanged before and after abrasion. Similar trends were noted for the Rq parameter, which represents the root mean square of the roughness profile ordinates (Figure 10). Notably, only the twill weave variant showed an increase in Rq value after abrasion—approximately 30% higher than the value before abrasion.

The Ra parameter—an arithmetic mean of the roughness profile ordinates—is a widely used and often prioritized metric in surface evaluation across various objects. In the context of textile materials, the Ra is commonly measured using the KES-FB4 instrument, one of the modules of the Kawabata Evaluation System (KES), which was developed for objective, instrumental assessment of fabric hand. It is a contact method and provides surface roughness data in two-dimensional scale. The sensitivity of measurement is significantly lower than that provided by non-contact optical methods. While suitable for evaluating fabric hand due to its correlation with the tactile sensitivity of human fingers, the KES-FB4’s measurement sensitivity is significantly lower than that offered by non-contact optical methods. As a result, it falls short when detailed analysis of surface changes due to abrasion is required. Furthermore, the Ra parameter alone does not provide sufficient information about the nature of the surface or the specific types of irregularities and alterations it undergoes.

If the analysis of the fabric surface before and after abrasion were based solely on the Ra parameter, one might conclude that the surface did not undergo significant changes due to abrasion, with the exception of the 3/1S twill weave fabric. However, such a conclusion would not accurately reflect the actual condition of the fabrics. Only a more comprehensive analysis, incorporating additional parameters that characterize the geometric structure of the fabric surface, enables a reliable assessment of surface changes caused by the abrasion process using the Martindale device. This is discussed below.

**Figure 11:**
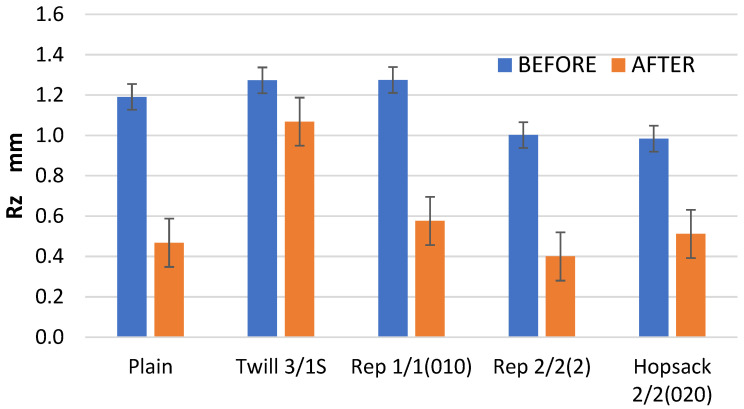
The values of the Rz parameter for the fabrics before and after the abrasion test.

The maximum height of the profile (Rz) was significantly reduced as a result of the abrasion cycles (Figure 11). This is in line with expectations, as the highest picks of the abraded surface are typically the first to wear down under abrasion. In general, the abrasion process leads to surface flattening, reflecting either abrasive or fatigue wear mechanisms. Unlike the Ra and Rq parameters, the Rz values clearly indicate the extent of surface wear caused by abrasion. The most substantial changes—approximately a 60% reduction in Rz compared to the pre-abrasion values –were noted in fabrics with plain and 2/2 (2) rep weaves. These were also the variants with the highest kurtosis (Rku) value before abrasion (Table 3), indicating the sharpest distribution of surface heights. In contrast, the smallest change in the Rz, around 16%, was observed in the 3/1S twill weave fabric. This is likely due to the long interlacing—the longest among all investigated fabric variants—on the surface of the twill weave fabric. These extended thread segments, arranged horizontally, allowed the abradant to glide over the surface more smoothly, minimizing peak cutting and reducing wear.

**Figure 12:**
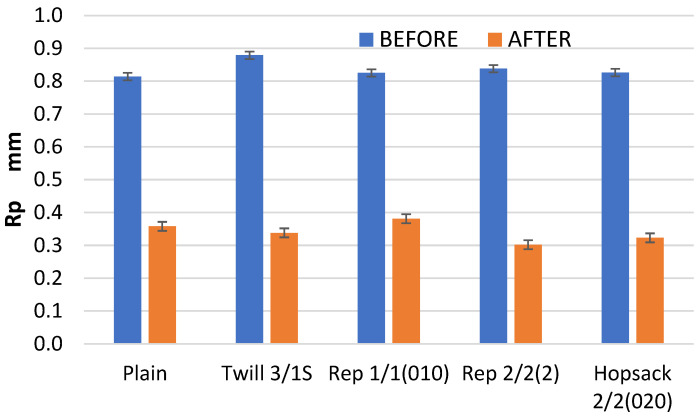
The values of the Rp parameter for the fabrics before and after the abrasion test.

The changes in the geometric structure of the fabric surface after 10,000 abrasion cycles are clearly illustrated by the Rp parameter (Figure 12), which represents the height of the highest pick of the profile within the sampling length. In all cases, the Rp value was significantly reduced, with changes ranging from 54% to 64% of the initial (pre-abrasion) Rp value.

**Figure 13:**
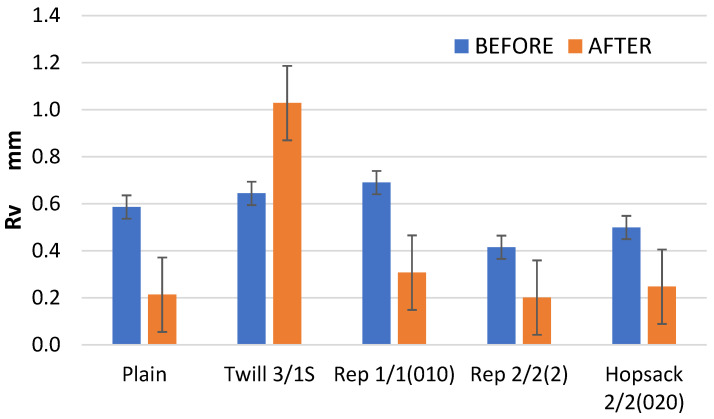
The values of the Rv parameter for the fabrics before and after the abrasion test.

An interesting phenomenon was observed in the case of the Rv parameter, which represents the depth of the lowest valley in the surface profile. As expected, in most cases, this depth was significantly reduced—by more than 50% (Figure 13). However, for the 3/1S twill weave fabric, a 60% increase in depth of the maximum valley of the roughness profile was observed. In the case of the 3/1S twill fabric, the fabric destruction process probably began due to the 10,000 abrasion cycles, resulting in one or more localized areas of damage, where dipper surface valleys formed. These changes in the Rv parameter for the 3/1S twill weave fabric explain the previously noted changes in the Rz parameter (Figure 11), which is defined as the sum of Rp and Rv.

**Figure 14:**
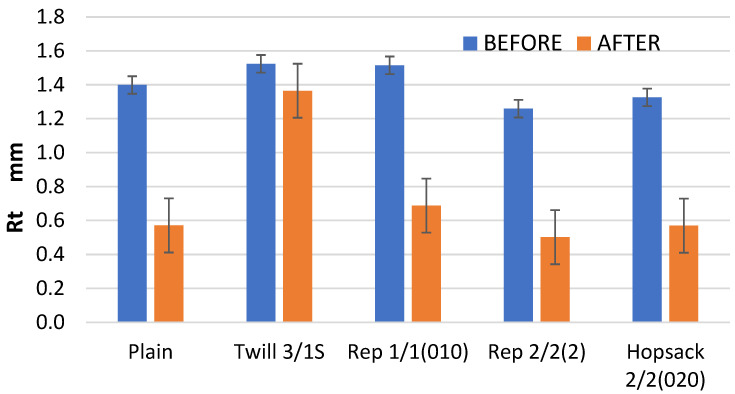
The values of the Rt parameter for the fabrics before and after the abrasion test.

Similar trends were noted for the Rt parameter, which represents the total height of the profile (Figure 14). In all cases, the Rt values after the abrasion test were lower than those recorded before the abrasion process. The smallest decrease was noted for the 3/1S twill fabric.

Kurtosis (Rku) is another surface geometry parameter that showed significant changes as a result of the fabric abrasion process. Kurtosis is a measure of the concentration of results around the mean value. An Rku value of 3 indicates a normal distribution; values greater than 3 reflect a sharp distribution, while values below 3 indicate a flattened distribution of height values (z-values).

**Figure 15:**
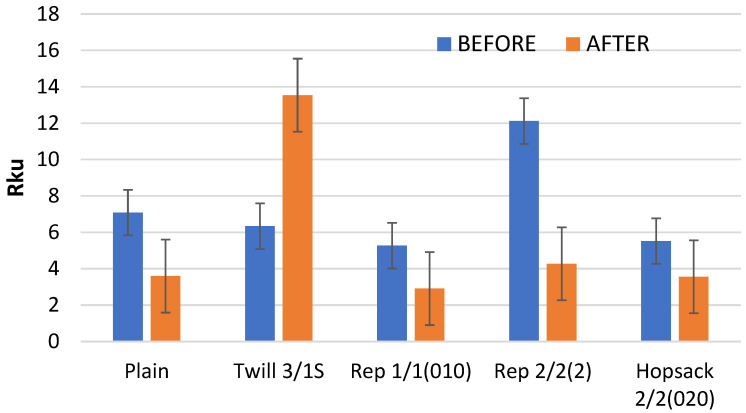
The values of the Rku parameter for the fabrics before and after the abrasion test.

For all analyzed fabric variants, the kurtosis values before abrasion were significantly higher than 3 (Figure 15), indicating a sharp distribution of point heights on the fabric surface. After abrasion, the kurtosis decreased for all variants except the 3/1S twill weave. Notably, only the 1/1 (010) rep weave fabric exhibited a post-abrasion kurtosis below 3—specifically, 2.91—indicating a slight flattening of the points’ height distribution on the fabric surface. Since this value is very close to 3, the distribution can be considered approximately normal. In contrast, the 3/1S twill weave fabric showed a dramatic increase in kurtosis—from 6.34 to 13.54—indicating a significant sharpening of the points’ height distribution on the fabric surface following abrasion.

The values of the Rku parameter are consistent with the histograms of surface height distribution presented later in the article.

Statistical analysis of data for the fabrics before the abrasion test confirmed that, in most cases, the weave significantly influenced the profilometer results. Exceptions were observed for two parameters: Rp and Rt, where the difference between the value of the parameters for fabrics of different weaves were statistically insignificant. The results of the one-way ANOVA for fabrics before the abrasion test are presented in the Supplementary Materials—Table S1.

For fabrics after the Martindale abrasion process, statistically significant differences between the fabrics of different weaves were observed for the majority of parameters. However, the influence of weave was found to be statistically insignificant for the Ra, Rp, and Rpk parameters. The results of the one-way ANOVA for fabrics after the abrasion test are presented in the Supplementary Materials—Table S2.

The differences in the parameter values obtained from the profilometer for fabrics before and after the Martindale test were evaluated using two-way ANOVA. Surface parameters were treated as the dependent variables, while weave type and “before/after” abrasion were treated as the main factors. The analysis revealed that, in most cases, statistically significant differences existed between the fabrics before and after the Martindale test. However, for the Ra and Rq parameters, the differences were not statistically significant at the 0.05 significance level. This supports our earlier conclusion that Ra and Rq are not suitable for assessing changes in fabric surface geometry caused by abrasion. Consequently, contact (stylus-based) surface measurement methods also appear inadequate for this purpose. The results of the two-way ANOVA are provided in the Supplementary Materials—Table S3.

**Figure 16:**
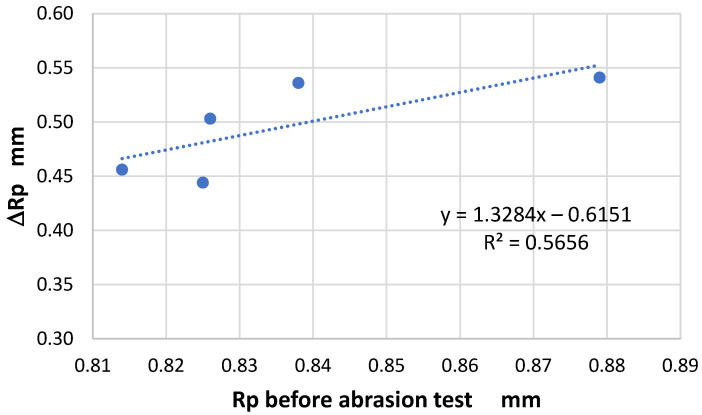
The relationship between the initial value of the Rp parameter and the changes in the parameter after 10,000 abrasion cycles.

The analysis further confirmed that the effect of the abrasion on the geometric structure of the fabric surface varied depending on fabric weave. In one specific case—the Rp parameter—it was found that the changes due to the abrasion were related to the initial (pre-abrasion) value of the parameter (Figure 16). For the other parameters, this relationship was weaker than that for the Rp parameter. This was largely due to the unique behavior of the 3/1S twill weave fabric, which differed from the other tested variants.

**Figure 17:**
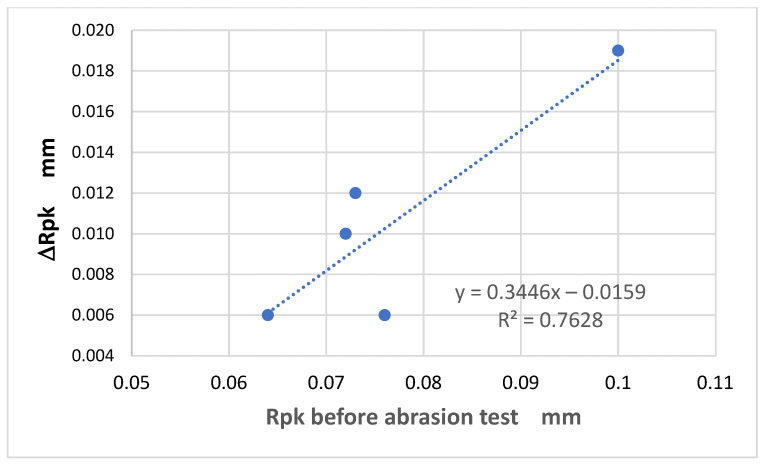
The relationship between the initial value of the Rpk parameter and the changes in the parameter after 10,000 abrasion cycles.

An interesting relationship was found for the Rpk parameter (Figure 17), which represents the reduced peak height—the average height of peaks above the core surface. This parameter is derived from the material ratio curve, also known as the Abbott–Firestone curve, which illustrates the percentage of material visible while slicing through a surface at various depths. It is generally accepted that the surface of solid objects consists of three components: core (central), peaks, and valleys. Accordingly, three parameters are used to characterize the surface heights: core roughness depth Rk (which describes the central part of the material ration curve), reduced peak height Rpk, and reduced valley depth Rvk [23]. Data provided by the profilometer showed that the Rpk parameter was particularly useful for analyzing fabric surface changes resulting from abrasion. Notably, the extent of change in Rpk after 10,000 cycles of abrasion was strongly correlated with its initial value. The correlation coefficient between the initial Rpk value and the change observed after abrasion was high, at Rx,y = 0.87 (Figure 17).

**Figure 18:**
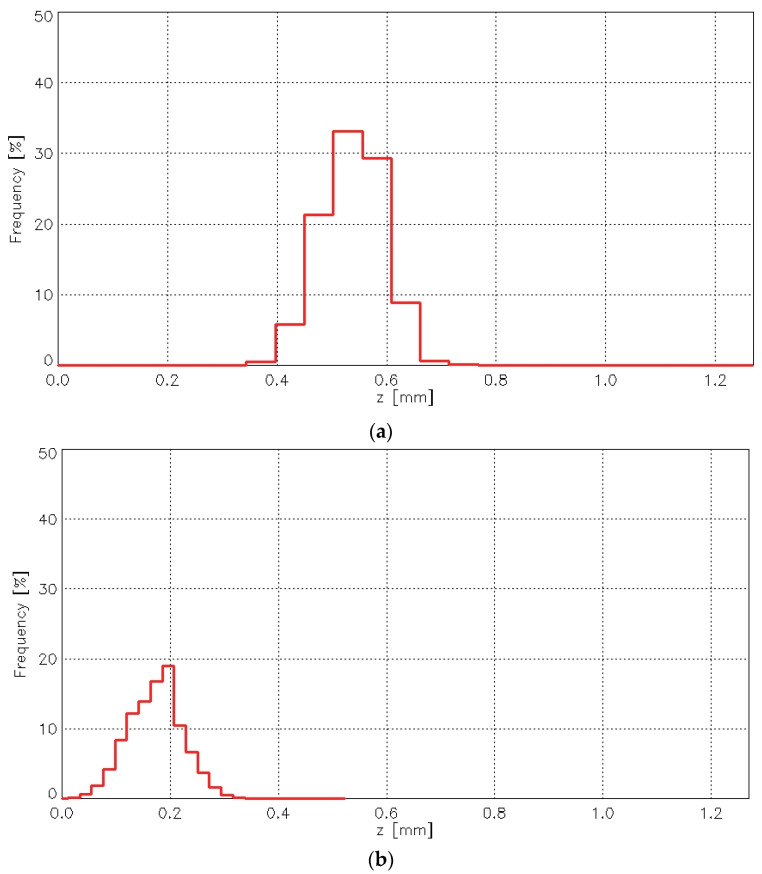
Histograms of the point height for the plain woven fabric: (**a**) before abrasion the test, (**b**) after the abrasion test.

The abrasion test caused significant changes in the height distribution of points on the fabric surface. Figure 18 presents histograms of the point height (z-values) for the plain weave fabric before (Figure 18a) and after (Figure 18b) the abrasion test.

**Figure 19:**
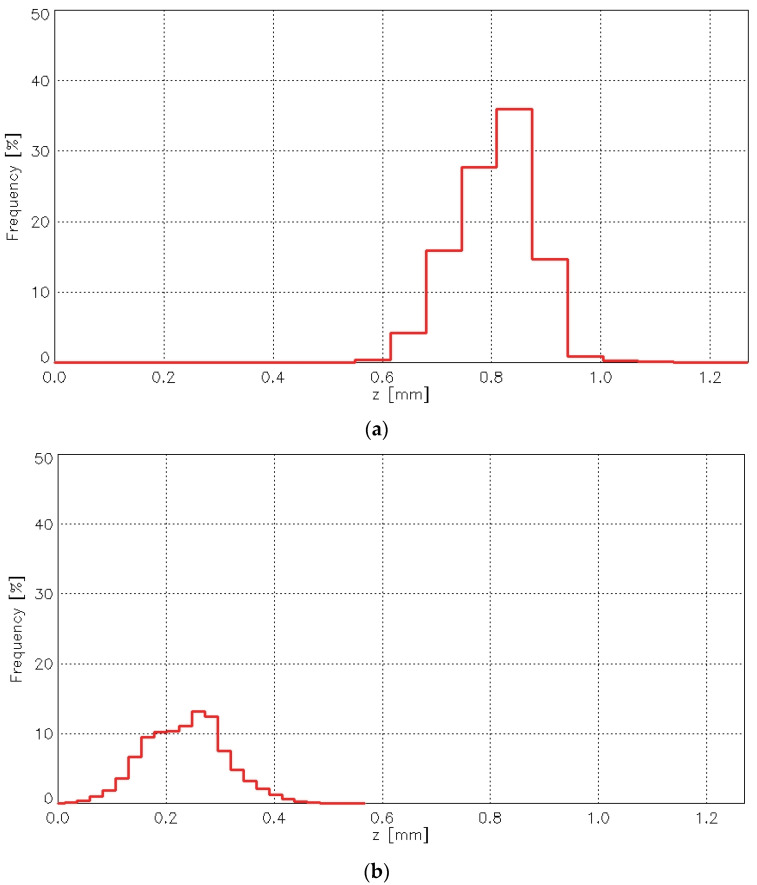
Histograms of the point height for the 1/1 (010) rep woven fabric: (**a**) before the abrasion test, (**b**) after the abrasion test.

It is clear that the changes affected both the most frequent height values and the histogram maximum—the position of the histogram peak. Similar changes were observed for the other investigated fabric variants. Figure 19 presents histograms for the 1/1 (010) rep weave fabric.

The changes in the parameters characterizing the height distribution of the fabrics are presented in Table 4.

For all fabric variants, the histogram maximum before the abrasion test was significantly higher than after the abrasion test. Similarly, the most frequent height values were greater before abrasion than after, with one exception—the 3/1S twill weave fabric. The abrasion test caused the histogram to flatten as a result of the removal of the highest surface points.

Analysis of all histograms representing the distribution of surface heights confirmed that abrasion caused substantial changes in the geometric structure of the fabric surface. Moreover, the results revealed that these changes varied depending on the fabric weave. Differences were also observed between samples of the same variant, suggesting possible inconsistencies in the surface geometry. This may have been influenced by the orientation of the fabric samples within the Martindale device during testing—a factor that warrants further, in-depth investigation.

**Figure 20:**
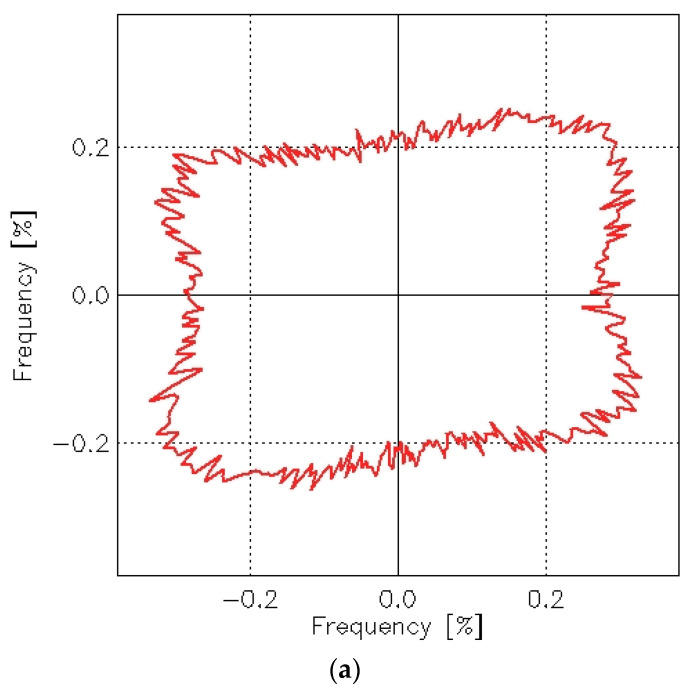

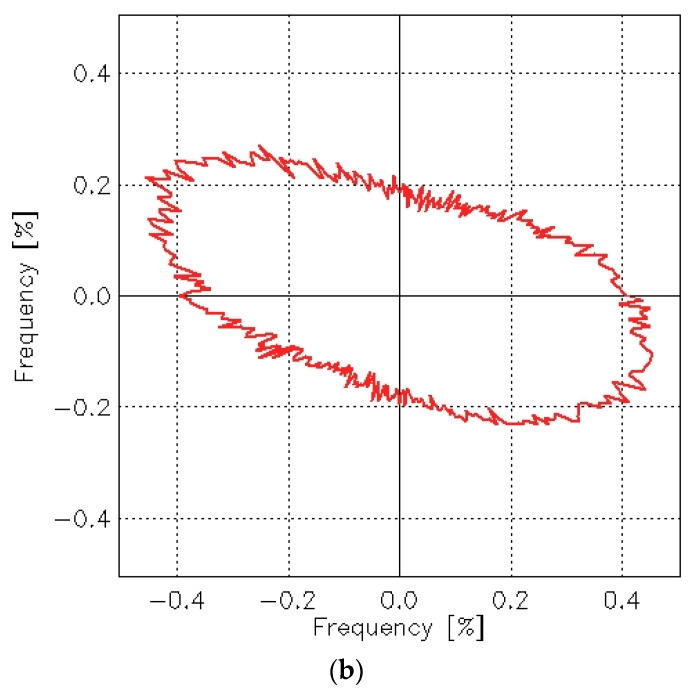
Angle distribution functions for the plain woven fabric: (**a**) before the abrasion test, (**b**) after the abrasion test.

The abrasion process also caused changes in texture direction, which can be identified by analyzing the angle distribution function. This function determines the orientation of surface slopes and can be represented in a polar coordinate plot (R-φ plot). The φ angle describes the direction normal to the edges and is measured counterclockwise starting at the positive *x*-axis. The radius R describes the frequency (in percent) of edges oriented in the φ direction. Angle distribution functions for the plain woven fabric before and after the abrasion test are shown in Figure 20.

It is evident that the abrasion test affected the texture direction of the plain woven fabric. Before abrasion, the fabric exhibited an isotropic surface—its geometric structure showed no clear directional dependence. After abrasion, however, a distinct directionality emerged in the surface geometry. Skewness appeared in the S direction on the angle distribution graph for the fabric after abrasion. This indicates that the texture direction post-abrasion was oriented in the Z direction. The angle distribution function reflects the orientation of tangents to the surface curvature at points of contact—specifically, lines that are perpendicular (at a 90° angle) to the radius at the point where the tangent meets the surface.

**Figure 21:**
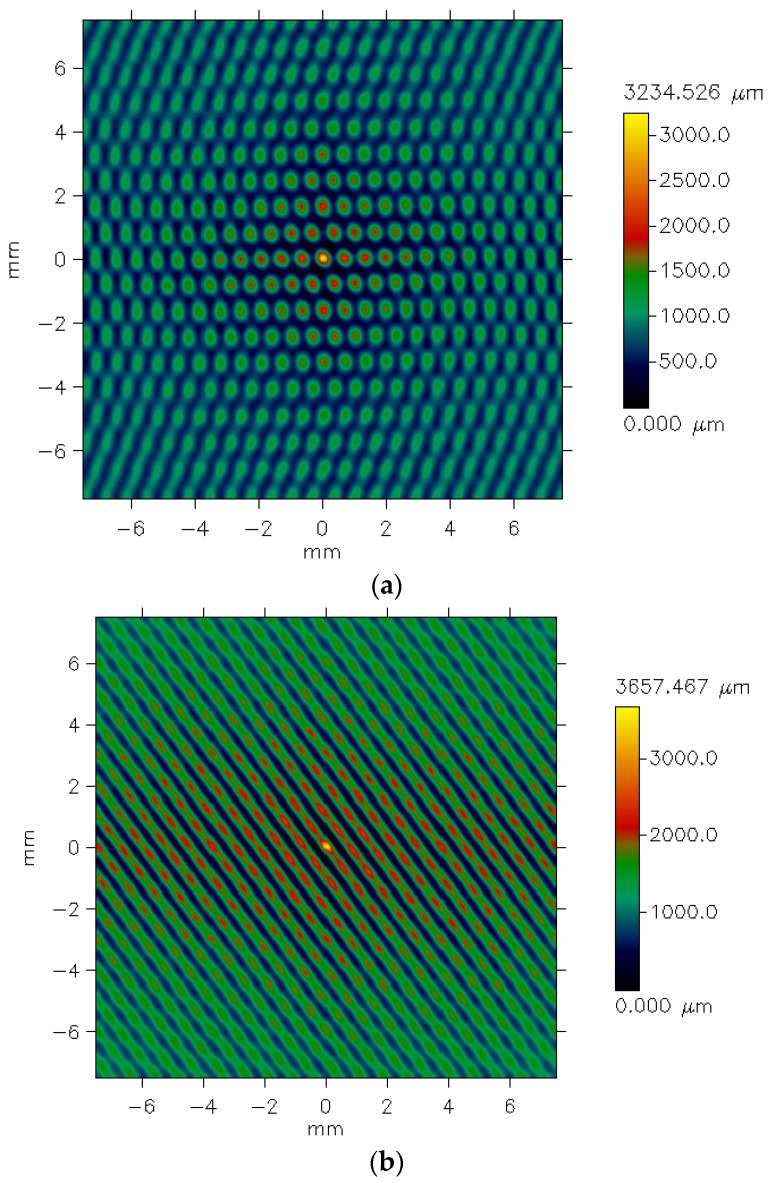
Autocorrelation functions for the plain woven fabric: (**a**) before the abrasion test, (**b**) after the abrasion test.

This observation aligns with the visual appearance of the fabric after abrasion, as shown in Figure 4b. The S-direction surface texture of the plain weave fabric is further confirmed by the autocorrelation function presented in Figure 21.

**Figure 22:**
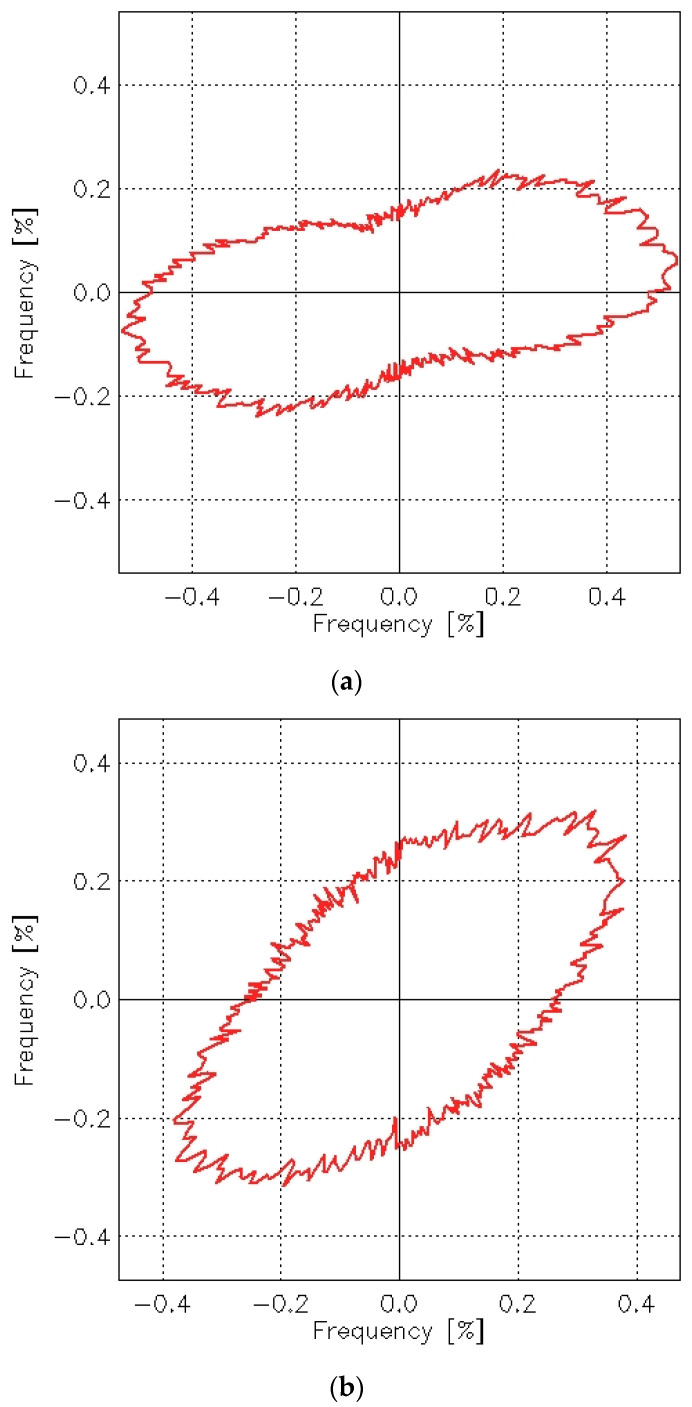
Angle distribution functions for the 3/1S twill woven fabric: (**a**) before the abrasion test, (**b**) after the abrasion test.

**Figure 23:**
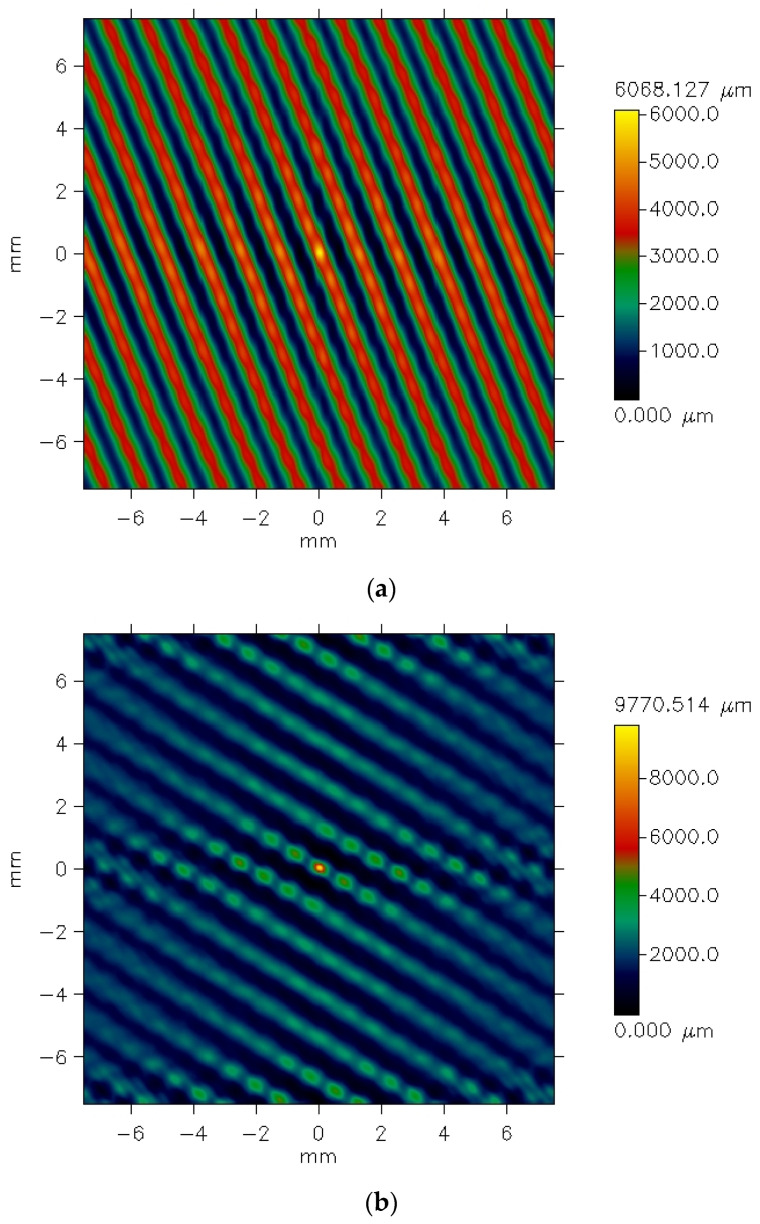
Autocorrelation functions for the 3/1S twill woven fabric: (**a**) before the abrasion test, (**b**) after the abrasion test.

In the case of the 3/1S twill weave fabric, the directionality of the surface texture was visible even before abrasion (Figure 22a). This was obvious, as the diagonal stripes characterizing the twill weave were visible even to the naked eye. Such directionality is a typical feature of fabrics with different twill weaves. After the abrasion test, the angle of these stripes changed, but the directional pattern remained clearly visible in both the angle distribution function (Figure 22b) and the autocorrelation function (Figure 23b).

Histograms, autocorrelation functions, and angle distribution functions can be generated for individual specimens of each investigated fabric variant. However, it is not possible to create average graphs of these functions for a given fabric variant. A detailed analysis of the autocorrelation functions and angle distribution plots confirmed that changes in the directionality of the fabric surface texture as a result of the abrasion process occurred across all investigated fabric variants. The abrasion process using the Martindale device either introduced or intensified the directionality of the fabric surface. For the rep and hopsack weave fabrics—as with the plain weave—no distinct surface directionality was observed before abrasion. However, slight directionality emerged following the abrasion process. In the case of the twill fabric, the abrasion process altered the orientation of the characteristic surface stripes.

The observed change in directionality of the fabric surface may stem from the test method itself. In the Martindale abrasion test, the fabric is rubbed against an abrasive medium in a translational motion that traces a Lissajous figure. This movement is repeated many times based on the number of abrasion cycles. Although this pattern is intended to distribute motion evenly and avoid dominance in any single direction, our results suggest that a directional bias still emerged. Further investigation is therefore warranted to understand how Martindale abrasion affects surface directionality and to determine whether this effect intensifies as the number of abrasion cycles increases.

It is important to note that this study focused on cotton woven fabrics made from the same yarns: 50 tex CO OE in the warp and 100 tex CO OE in the weft. The nominal warp and weft densities were adjusted to be identical across all fabric variants. Additionally, all fabrics were manufactured on the same loom and underwent the same finishing process. This allowed for isolated assessment of the influence of weave on the abrasion behavior of the fabrics. However, factors such as the raw material composition, the way of finishing, and the fabric density could not be evaluated in this context, as they remained constant across the samples. At present, it is difficult to draw definitive conclusions due to the influence of the so-called “fabric phase” [9,10]. After removal from the loom and during the finishing process, yarns undergo relaxation, which subtly alters the fabric structure. This can particularly affect the height and distribution of extreme surface points relative to the mean surface plane, as confirmed by the histograms of the point heights on the fabric surfaces. This characteristic warrants further investigation.

Our research clearly confirmed the importance of interlacing points per length unit and length of thread floats in determining the abrasion resistance of woven fabrics. The most significant surface changes due to the abrasion process were observed in the 3/1S twill woven fabric, which had the fewest number of interlacing points and the longest thread floats of all investigated fabrics. These findings are consistent with the results reported by Kaynak, H.K et al. [3] and Collier [5].

## 5. Conclusions

Based on the conducted research and the obtained results, it can be stated that measurements of the geometric structure of fabric surfaces using a non-contact optical method allow for the evaluation of changes in the structure of the surface subjected to the abrasion process using the Martindale device. After 10,000 abrasion cycles, notable changes were observed in the values of nearly all parameters characterizing the surface geometry of the fabrics. Depending on the parameter, these values either increased or decreased. Based on these results, it can be assumed that certain surface geometry parameters—such as the maximum height of the roughness profile (Rz), the height of the highest peak (Rp), the depth of the deepest valley (Rv), the total roughness height (Rt), and kurtosis (Rku)—can be effectively used to assess the abrasion resistance of fabrics. However, the practical application of these parameters requires further research, including studies involving various numbers of abrasion cycles, analysis of additional changes in the abraded samples (e.g., mass loss), and correlation of these changes with surface geometry parameters.

Unfortunately, the obtained results also indicate that the most common and routinely determined parameters—the arithmetic mean of the roughness profile ordinates (Ra) and the root mean square of the roughness profile ordinates (Rq)—are not suitable for assessing abrasion resistance. While slight changes in these parameters were observed after abrasion, they were generally insignificant and did not follow a clear trend across fabric variants. Although they reflected some level of surface change, they failed to characterize the nature of those changes meaningfully.

In addition, non-contact optical measurement allows for the assessment of surface texture directionality. For this purpose, the autocorrelation function and angle distribution function can be applied. These tools provide insight into the directional characteristics of the fabric surface before and after abrasion.

In conclusion, the use of the non-contact optical measurement for evaluating the abrasion resistance of textile materials shows promise but requires further investigations. The presented study is preliminary and was conducted to confirm the potential of the 3D contactless optical measurement of surface geometry in analyzing abrasion-induced changes in fabrics. The results presented here form a foundation for future research aimed at developing an objective, instrumental approach to assessing surface changes following abrasion.

## Figures and Tables

**Table 2:** The results from the profilometer.

Fabric Variant		Ra (SD) mm	Rq (SD) mm	Rz (SD) mm	Rp (SD) mm
Plain	Before abrasion	0.044 (0.00090)	0.056 (0.0007)	1.191 (0.1354)	0.814 (0.1284)
	After abrasion	0.042	0.053	0.468	0.358
Twill	Before abrasion	0.053 (0.0013)	0.068 (0.0018)	1.273 (0.0888)	0.879 (0.0758)
	After abrasion	0.077	0.115	1.068	0.338
Rep 1/1 (010)	Before abrasion	0.060 (0.0019)	0.075 (0.0025)	1.275 (0.8652)	0.825 (0.0844)
	After abrasion	0.063	0.078	0.576	0.381
Rep 2/2 (2)	Before abrasion	0.036 (0.0005)	0.047 (0.0029)	1.002 (0.1004)	0.838 (0.0332)
	After abrasion	0.031	0.042	0.400	0.302
Hopsack	Before abrasion	0.050 (0.0011)	0.053 (0.0015)	0.984 (0.0580)	0.826 (0.0677)
	After abrasion	0.050	0.053	0.512	0.323

**Table 3:** Continuation of the results from the profilometer.

Fabric Variant		Rv (SD) mm	Rt (SD) mm	Rpk (SD) mm	Rku (SD) -
Plain	Before abrasion	0.586 (0.0355)	1.399 (0.1341)	0.064 (0.0081)	7.087 (1.6761)
	After abrasion	0.213	0.571	0.058	3.593
Twill	Before abrasion	0.644 (0.0673)	1.524 (0.1132)	0.072 (0.0053)	6.338 (0.7230)
	After abrasion	1.028	1.365	0.062	13.540
Rep 1/1 (010)	Before abrasion	0.690 (0.0844)	1.515 (0.0631)	0.100 (0.0143)	5.271 (0.4752)
	After abrasion	0.307	0.688	0.081	2.908
Rep 2/2 (2)	Before abrasion	0.415 (0.0332)	1.259 (0.2210)	0.073 (0.0029)	12.117 (1.8714)
	After abrasion	0.201	0.502	0.061	4.270
Hopsack	Before abrasion	0.499 (0.0677)	1.326 (0.2298)	0.076 (0.0040)	5.521 (0.4130)
	After abrasion	0.247	0.570	0.070	3.556

**Table 4:** Average histogram parameters for fabrics before and after the abrasion test.

Fabric Variant	Most Frequent Height mm		Histogram Maximum %	
	Before Abrasion	After Abrasion	Before Abrasion	After Abrasion
Plain	0.567	0.214	39.19	19.00
Twill 3/1S	0.647	1.054	44.55	34.36
Rep 1/1 (010)	0.695	0.316	32.41	15.18
Rep 2/2 (2)	0.391	0.198	45.47	27.51
Hopsack 2/2 (020)	0.507	0.270	36.42	20.14

## Data Availability

The original contributions presented in this study are included in the article/Supplementary Material. Further inquiries can be directed to the corresponding author.

## Supplementary Materials

The following supporting information can be downloaded at: https://www.mdpi.com/article/10.3390/ma18153636/s1, Figure S1. The exemplary pictures of the investigated pain weave fabric before and after the abrasion test: (a) before abrasion, (b) after abrasion. Figure S2. The exemplary pictures of the investigated 3/1S twill weave fabric before and after the abrasion test: (a) before abrasion, (b) after abrasion. Figure S3. The exemplary pictures of the investigated rep 1/1 (010) rep weave fabric before and after the abrasion test: (a) before abrasion, (b) after abrasion. Figure S4. The exemplary pictures of the investigated 2/2 (2) rep weave fabric before and after the abrasion test: (a) before abrasion, (b) after abrasion. Figure S5. The exemplary pictures of the investigated 2/2 (020) hopsack weave fabric before and after the abrasion test: (a) before abrasion, (b) after abrasion. Table S1. One-way ANOVA results for the fabrics before Martindale tests. Table S2. One-way ANOVA results for the fabrics after Martindale tests. Table S3. Two-way ANOVA for fabrics before and after Martindale tests. Table S4. Individual results for fabrics after abrasion test.
