# Supplementary material for: Quantitative Assessment of Woven Fabric Surface Changes During Martindale Abrasion Using Contactless Optical Profilometry
Source: Materials (Basel). 2025 Aug 1;18(15):3636. doi: 10.3390/ma18153636 (PMC12348827; doi:10.3390/ma18153636)
Supplement: Supplementary file 1 [file materials-18-03636-s001.zip › materials-3754477-supplementary.pdf]

a) 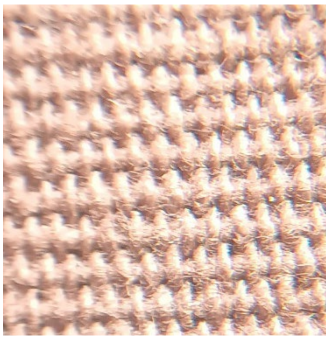 b) 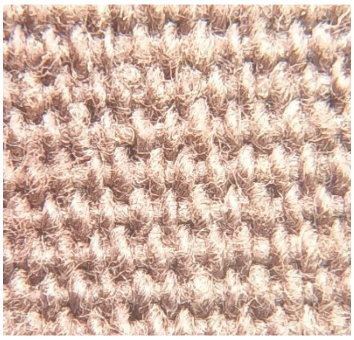

Figure S1. The exemplary pictures of the investigated pain weave fabric before and after the abrasion test: (a) before abrasion, (b) after abrasion.

a) 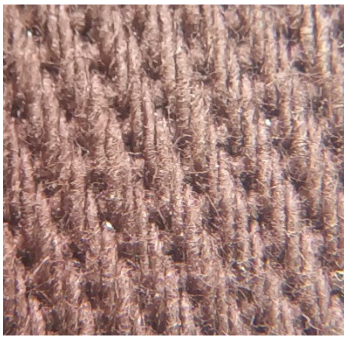 b) 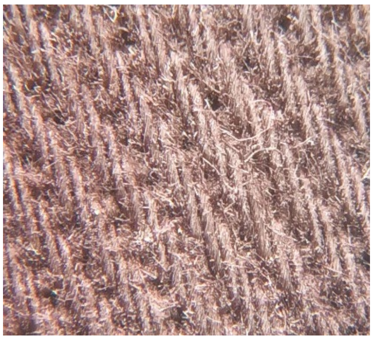

Figure S2. The exemplary pictures of the investigated 3/1 S twill weave fabric before and after the abrasion test: (a) before abrasion, (b) after abrasion.

a) 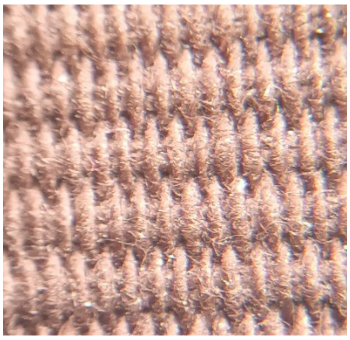 b) 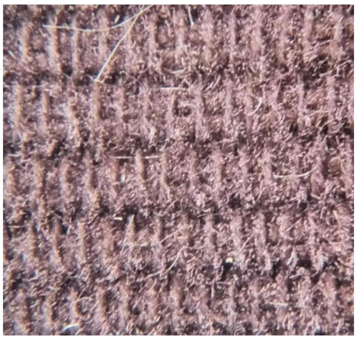

Figure S3. The exemplary pictures of the investigated rep 1/1 (010) rep weave fabric before and after the abrasion test: (a) before abrasion, (b) after abrasion.

a) 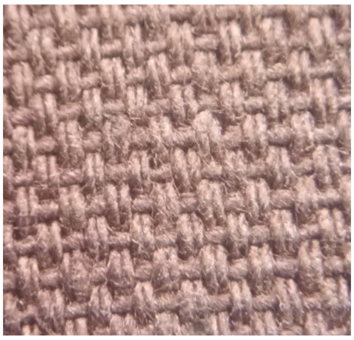 b) 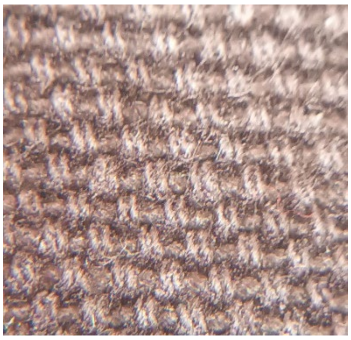

Figure S4. The exemplary pictures of the investigated 2/2 (2) rep weave fabric before and after the abrasion test: (a) before abrasion, (b) after abrasion.

a) 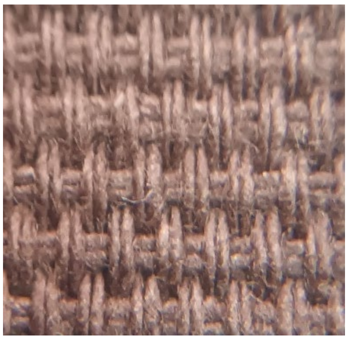 b) 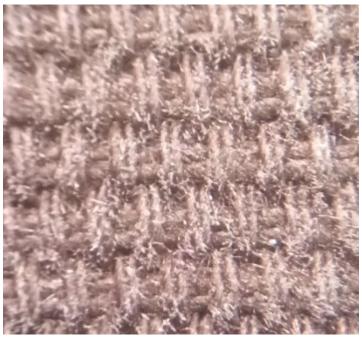

Hopsack 2/2 (020) weave fabric

Figure S5. The exemplary pictures of the investigated 2/2 (020) hopsack weave fabric before and after the abrasion test: (a) before abrasion, (b) after abrasion.

Table S1. One-way ANOVA results for the fabrics before Martindale tests.

Parameter	One-way ANOVA results							
	SS Effect	df Efect	MS Efect	SS Error	df Error	MS Error	F	p
Ra	0.0017	4	0.00043	0.00003	20	0.000002	271.6076	0.000000
Rq	0.0024	4	0.00059	0.00008	20	0.000004	142.5778	0.000000
Rz	0.4100	4	0.10250	0.18858	20	0.009429	10.8709	0.000076
Rp	0.0130	4	0.00324	0.14021	20	0.007011	0.4620	0.762731
Rt	0.2704	4	0.06761	0.54573	20	0.027287	2.4778	0.077050
Rku	157.2630	4	39.31575	28.92163	20	1.446082	27.1878	0.000000
Rv	0.2856	4	0.07141	0.07441	20	0.003721	19.1919	0.000001
Rpk	0.0038	4	0.00095	0.00129	20	0.000065	14.7426	0.000009

Legend: SS – sum of squares, df – degree of freedom, MS – mean square, F – variable of F distribution, p – statistical significance.

Table S2. One-way ANOVA results for the fabrics after Martindale tests.

Paraeter	One-way ANOVA results							
		df Efect	MS Efect	SS Error	df Error	MS Error	F	p
Ra	0.0026	4	0.00064	0.00078	5	0.000156	4.1093	0.076636
Rq	0.0064	4	0.00160	0.00123	5	0.000246	6.5020	0.032334
Rz	0.5691	4	0.14227	0.05256	5	0.010512	13.5333	0.006837
Rp	0.0076	4	0.00189	0.00898	5	0.001796	1.0527	0.465261
Rt	1.0155	4	0.25388	0.02332	5	0.004663	54.4465	0.000260
Rku	160.5171	4	40.12926	35.13699	5	7.027398	5.7104	0.041700
Rv	1.0011	4	0.25027	0.00807	5	0.001614	155.0789	0.000020
Rpk	0.0007	4	0.00017	0.00019	5	0.000038	4.4947	0.065284

Legend: SS – sum of squares, df – degree of freedom, MS – mean square, F – variable of F distribution, p – statistical significance.

Table S3. Two-way ANOVA for fabrics before and after Martindale tests.

Ra					
Effect	SS	df	MS	F	p
Intercept	0.072518	1	0.072518	1328.689	0.000000
Weave	0.003501	4	0.000875	16.036	0.000000
Before/after	0.000095	1	0.000095	1.734	0.198212
Error	0.001583	29	0.000055		
**Rq**					
Intercept	0.123922	1	0.123922	899.8181	0.000000
Weave	0.006084	4	0.001521	11.0435	0.000014
Before/after	0.000444	1	0.000444	3.2238	0.083002
Error	0.003994	29	0.000138		
**Rz**					
Intercept	21.86750	1	21.86750	1275.025	0.000000
Weave	0.72285	4	0.18071	10.537	0.000021
Before/after	2.08826	1	2.08826	121.760	0.000000
Error	0.49737	29	0.01715		
**Rp**					
Intercept	9.886466	1	9.886466	1784.468	0.000000
Weave	0.009048	4	0.002262	0.408	0.801152
Before/after	1.760660	1	1.760660	317.792	0.000000
Error	0.160668	29	0.005540		
**Rt**					
Intercept	32.82035	1	32.82035	919.9845	0.000000
Weave	0.82046	4	0.20512	5.7496	0.001559
Before/after	3.16407	1	3.16407	88.6918	0.000000
Error	1.03457	29	0.03567		
**Rku**					
Intercept	1177.615	1	1177.615	144.3569	0.000000
Weave	145.266	4	36.317	4.4518	0.006298
Before/after	20.493	1	20.493	2.5121	0.123819
Error	236.572	29	8.158		
**Rv**					
Intercept	9.330472	1	9.330472	249.5781	0.000000
Weave	0.285002	4	0.071250	1.9059	0.136240
Before/after	0.849784	1	0.849784	22.7306	0.000048
Error	1.084164	29	0.037385		
**Rpk**					
Intercept	0.146187	1	0.146187	2541.396	0.000000
Weave	0.004314	4	0.001078	18.748	0.000000

Before/after	0.000842	1	0.000842	14.645	0.000639
Error	0.001668	29	0.000058		

Table S4. Individual results for fabrics after abrasion test.

Weave	Ra [mm]	Rq [mm]	Rz [mm[	Rp [mm]	Rv [mm]	Rt [mm]	Rku [-]	Rpk [mm]
Plain	0.040	0.050	0.427	0.338	0.185	0.523	3.220	0.050
	0.044	0.056	0.508	0.377	0.241	0.618	3.966	0.066
Twill 3/1 S	0.057	0.090	0.915	0.322	0.983	1.305	17.708	0.064
	0.096	0.139	1.220	0.353	1.072	1.425	9.371	0.059
Rep 1/1 (010)	0.061	0.076	0.546	0.353	0.274	0.627	2.857	0.078
	0.064	0.079	0.606	0.409	0.340	0.749	2.958	0.083
Rep 2/2 (2)	0.032	0.043	0.399	0.304	0.208	0.512	4.142	0.062
	0.029	0.040	0.400	0.299	0.194	0.492	4.398	0.059
Hopsack 2/2 (2)	0.049	0.062	0.534	0.378	0.235	0.614	3.740	0.074
	0.050	0.063	0.490	0.267	0.258	0.525	3.371	0.066
